# Mental Imagery: Functional Mechanisms and Clinical Applications

**DOI:** 10.1016/j.tics.2015.08.003

**Published:** 2015-10

**Authors:** Joel Pearson, Thomas Naselaris, Emily A. Holmes, Stephen M. Kosslyn

**Affiliations:** 1 School of Psychology, The University of New South Wales, Sydney, Australia; 2 Department of Neuroscience, Medical University of South Carolina, Charleston, SC, USA; 3 Medical Research Council Cognition and Brain Sciences Unit, Cambridge, UK; 4 Department for Clinical Neuroscience, Karolinska Institutet, Stockholm, Sweden; 5 Minerva Schools at the Keck Graduate Institute, San Francisco, CA, USA

## Abstract

Mental imagery research has weathered both disbelief of the phenomenon and inherent methodological limitations. Here we review recent behavioral, brain imaging, and clinical research that has reshaped our understanding of mental imagery. Research supports the claim that visual mental imagery is a depictive internal representation that functions like a weak form of perception. Brain imaging work has demonstrated that neural representations of mental and perceptual images resemble one another as early as the primary visual cortex (V1). Activity patterns in V1 encode mental images and perceptual images via a common set of low-level depictive visual features. Recent translational and clinical research reveals the pivotal role that imagery plays in many mental disorders and suggests how clinicians can utilize imagery in treatment.

## Trends

Recent research suggests that visual mental imagery functions as if it were a weak form of perception.

Evidence suggests overlap between visual imagery and visual working memory – those with strong imagery tend to utilize it for mnemonic performance.

Brain imaging work suggests that representations of perceived stimuli and mental images resemble one another as early as V1.

Imagery plays a pivotal role in many mental disorders and clinicians can utilize imagery to treat such disorders.

## Mental Imagery

Mental imagery has played a central role in discussions of mental function for thousands of years. Many have argued that it is one of the primary human mental events that allow us to remember, plan for the future, navigate, and make decisions. In addition, mental imagery plays a core role in many mental health disorders and plays an increasingly important role in their treatment.

We use the term ‘mental imagery’ to refer to representations and the accompanying experience of sensory information without a direct external stimulus. Such representations are recalled from memory and lead one to re-experience a version of the original stimulus or some novel combination of stimuli. Note that not all mental imagery need be voluntary; external events or internal associations also can trigger a mental image, even if one does not want to experience the image at that time [1]. Mental imagery can clearly involve all of the senses, but in this review we focus on visual mental imagery, given that most empirical work has addressed this sensory domain.

Historically, mental imagery research suffered for both practical and theoretical reasons. Methodological constraints caused by imagery's inherently private nature put practical limits on the types of mechanistic investigation that could be performed. Furthermore, the second half of the 20th century saw the rise of behaviorism in psychology. This theoretical orientation rejected the study of internal representations, including mental imagery. The combination of these two impediments is largely responsible for the comparative lack of mental imagery research relative to related topics such as visual attention and visual working memory [2].

Such constraints are now lifting, with increasingly sophisticated research techniques leading to many new discoveries about imagery. In recent years, new objective research methods have permitted more direct investigations into the mechanisms and neural substrates of mental imagery. Results from these methods shed light on mental imagery's role in perception, cognition, and mental health. Findings have cemented our understanding of visual mental imagery as a depictive internal representation with strong and unexpected ties to visual perception, effectively ending the so-called ‘imagery debate’ [3]. Moreover, studies reveal that mental imagery plays a pivotal role in clinical disorders such as anxiety. This upsurge in fundamental and clinical science regarding mental imagery is revealing the central role that mental imagery plays in everyday behavior as well as in human mental function and dysfunction.

## Mental Imagery and Weak Perception

Much of the work on imagery and perception in the 1990s and 2000s revealed that imagery shares processing mechanisms with like-modality perception. For example, researchers showed that imagined visual patterns interact with a concurrent perceptual stimulus to boost sensory performance in a detection task [4]. Many studies converged in demonstrating that mental imagery could function much like afferent sensory perception. Imagining oriented lines can induce an orientation aftereffect [5] or imagining a moving stimulus can induce a motion aftereffect on a subsequent perceptual stimulus, much like normal perception [6].

Mental images can also take the place of perceptual stimuli during various types of learning. Perceptual learning typically involves repeated performance of a perceptual detection or discrimination task that leads to increases in performance. However, imagining the crucial components of such a task, instead of actually performing them on a perceptual stimulus, can also enhance performance on the perceptual task [7]. For example, when participants repeatedly imagine a vertical line between two perceptual lines they subsequently perform better in discriminating the distances between three perceptual lines [7]. Similarly, classical conditioning can occur with voluntarily formed visual imagery in place of perceptual stimuli [8]. In both of these examples the imagery-based learning is later tested with perceptual stimuli, which demonstrates generalization from the imagined to the perceptual content.

One important requirement in mental imagery research is to ensure that the effect of visual imagery on concurrent perception is not merely being driven by visual attention. Many studies have demonstrated that applying attention to a particular stimulus, or part of one, can change multiple dimensions of sensory perception. For example, attention alone can increase stimulus contrast, color, or coherency [9–11]. Studies using the ‘**binocular rivalry**’ technique (see Glossary) have demonstrated contrasting effects of imagery and attention. When participants visualize one of two patterns, the imaged pattern has a much higher probability of being perceptually dominant in a subsequent brief single binocular rivalry presentation [2,12,13]. In other words, the content of the mental image primes subsequent dominance in binocular rivalry, just as a weak or low-contrast perceptual stimulus would do. Moreover, these effects grow stronger with longer image generation times, whereas increasing periods of applying attention to a particular stimulus does not modulate this priming effect [12]. Furthermore, particular experimental manipulations can attenuate the priming effect of imagery while leaving intact the effect of prior attention [12]. Thus, imagery can be dissociated from visual attention along at least two different dimensions.

**Figure 1:**
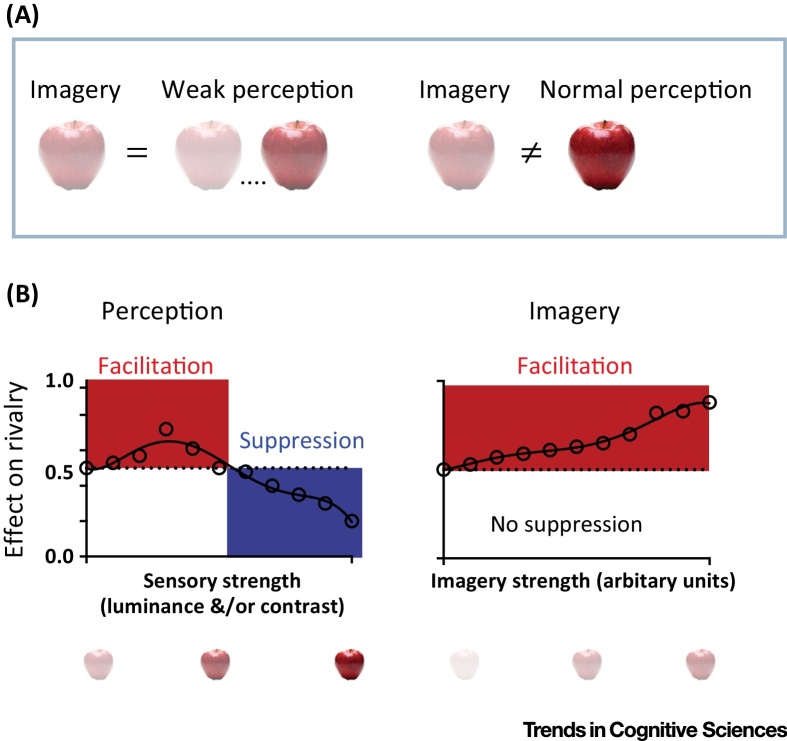
Imagery Resembles a Weak Version of Perception. (A) A useful way to conceptualize mental imagery is as a weak form of sensory perception. (B) A schematic illustration of the effects of prior perceptual stimuli at different strengths and of imagery on subsequent perception. The left graph shows hypothetical data for prior perceptual stimuli at different strengths (e.g., contrasts). Low-contrast prior stimulation facilitates subsequent detection [16] or binocular rivalry dominance [12,15], whereas high-contrast prior stimulation will induce a suppressive aftereffect. By contrast, on the right graph, imagery only facilitates subsequent perception. Overall, imagery acts much like weak perception. Schematic data plots are based on data from [12,15].

An emerging consensus from multiple behavioral studies is that the influence of prior perceptual stimuli on subsequent perceptual tasks depends on the ‘perceptual energy’ or strength of the prior stimulus [12,14–16]. Facilitation is more likely if the preceding stimulus is short and/or low contrast, whereas suppression is more likely when a prior stimulus is high contrast and/or is shown for a long duration [12,14–16]. Hence, the facilitative effect of a prior stimulus increases as the strength or presentation duration increases until it reaches a tipping point, when the effect reverses and leads to reduced facilitation and increased suppression (Figure 1) [12,15,16]. Evidence suggests a single continuous mechanism that depends on the visual ‘energy’ of the prior stimulus; that is, its sensory strength. Behavioral data thus far show that the effect of imagery on subsequent perception is limited to the facilitation range, and not suppression (Figure 1B, right panel) (for a review see [2]).

If mental imagery is conceived of as a type of top-down perception, visual features such as luminance or brightness should also be preserved in imagined representations and should have similar effects on physiology. Indeed a recent study demonstrated exactly that: the brightness of the imagined stimulus had a reliable and predictable effect on pupil constriction, as it does during perception [17].

**Figure 2:**
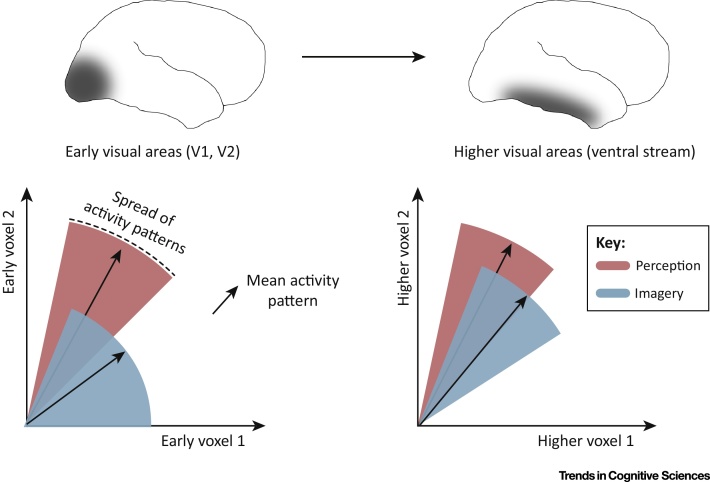
Activity Patterns Evoked by Visual Perception and Visual Mental Imagery Are Increasingly Similar with Ascension of the Processing Hierarchy. This diagram summarizes an organizing principle that is implicit in the fMRI literature on visual mental imagery. Here the ventral stream is coarsely grouped into early visual areas (shaded brain region, left panels) that represent low-level visual features (e.g., edges, textures) and higher-level visual areas (shaded region, right panels) that represent scene-level information and object categories. For purposes of illustration, we consider hypothetical multivoxel populations comprising just two voxels in the early visual cortex (left) and two voxels in the higher visual cortex (right). Activity patterns are represented as vectors in a 2D space in which the axes correspond to the two hypothetical voxels. In the early visual areas, activity associated with mental imagery has a lower signal-to-noise ratio (SNR) than activity associated with perception. This means that the mean activity vector (black arrow) evoked by visualizing a particular stimulus is shorter than the mean activity vector evoked by actually seeing a corresponding stimulus, while the spread of activity patterns around the mean activity vector (arc length) is larger. In the higher visual areas, the SNR associated with mental imagery is not as severely attenuated.

In addition, brain imaging work has provided compelling evidence that visual mental images arise from activation of the same types of visual features that are activated during visual perception. Several studies have explicitly modeled the representations encoded in activity during perception and then used the model to decode mental images from brain activity. To our knowledge this explicit modeling approach – known as **voxel**-wise modeling and decoding (VM) – was first applied to mental imagery in 2006 [18]. This landmark study designed a voxel-wise model of tuning to **retinotopic** location (i.e., a receptive field model) and then used it to decode mental images of high-contrast blobs in various configurations. Consistent with the evidence for retinotopic organization in mental imagery [19], models of retinotopic tuning during visual perception could be used to identify and even reconstruct mental images of the stimuli (Figure 2).

It could be argued that imagining simple, blob-like stimuli that are optimized to engage area V1 is a special case of mental imagery that may be unrelated to the rich, complex imagery we generate and use daily. However, the representation of **low-level visual features** is conserved even when people visualize complex, multi-object scenes such as photographs and artwork [20]. A model that was tuned to retinotopic location, spatial frequency, and orientation [21] picked out mental images of specific works of art from among thousands of other randomly selected images – and even from other examples of the artists’ own work. Performance was lower than that for actual perception, but still much better than that expected by chance. Thus, representations of retinotopy and spatial frequency – quintessential ‘visual’ representations – are encoded in activity even during rich and complex mental imagery.

Furthermore, sensitivity to both perceptual orientation and location in visual space has been linked to the anatomy of V1 [22,23]. Likewise, mental imagery precision of spatial orientation and location in retinotopic space are both associated with the size of V1 [24]. In fact, the precision of both mental imagery and visual perception is correlated with the size of area V1, providing further support for the commonalties between the two.

Together, these studies show that activity patterns in the visual cortex are not merely similar across visual mental imagery and perception: activity patterns encode a common set of visual representations. When considered in light of the behavioral evidence reviewed above, these results lend further support to the conceptualization of visual mental imagery as a weak or noisy form of top-down perception that can in some cases take the place of bottom-up perception.

## Mental Imagery and Visual Working Memory

Although mental imagery and visual working memory both involve the ability to represent and manipulate visual information, research on the two topics has diverged into two separate literatures that rarely reference one another [25]. Because of the different behavioral measures and tasks used, it has proved challenging to establish the degree of commonality between the two functions.

When participants in visual working memory experiments are asked to describe the strategies they use to complete the memory task, they tend to describe one of two different strategies. One involves creating a mental image to compare with the subsequent test stimuli [26–28]; the other strategy involves picking out particular details of a scene or array and encoding them phonologically or verbally, which is then compared with the test stimuli [26,28,29].

Recent behavioral work supports these subjective reports of different strategies [29,30] (but see [31]). This behavioral work directly compared the sensory strength of mental imagery and different measures of visual working memory. Individuals with stronger mental imagery had greater precision and higher capacity in visual working memory tasks, but not in iconic or verbal working memory tasks [29,30]. Furthermore, only those participants with strong sensory imagery were perturbed by the passive presence of uniform background luminance during visual working memory storage, but not in a verbal working memory task. Importantly, the creation of visual mental images is also perturbed by the presence of uniform passive luminance [12,32]. In addition, in a similar vein to visual imagery, the content of visual working memory can bias perception [33] and can facilitate detection in the neglected hemifield of visual extinction patients [34].

Taken together, these behavioral data suggest that those with relatively strong mental imagery utilize it to perform a visual working memory task whereas those with weaker imagery tend to rely on nonvisual strategies.

Brain imaging work has demonstrated overlap in the neural representation of visual working memory and mental imagery. For example, in one study [35], on some trials, participants were required to hold an oriented grating pattern in visual working memory until their memory performance was tested with a probe stimulus; on other trials, the same participants had to form and rotate a mental image of the same grating in accordance with a given cue. BOLD activity patterns in area V1 enabled accurate decoding of which pattern was being held in visual working memory and in the mental rotation (imagery) condition. When the classifier was trained on data from the working memory condition and then applied to decode data from the imagery condition, performance was just as high [35]. This generalization of decoding from memory to imagery is evidence for commonalities in the spatial pattern of BOLD activity during the two tasks. This in turn is evidence for representational overlap between mental imagery and visual working memory. Recent results also show that both visual working memory capacity and imagery strength and precision are associated with the surface size of V1 [24,36].

The combination of behavioral and brain imaging data shows that, despite clear task differences (‘Hold this visual information in memory and we will subsequently test you on it’ vs ‘Create a mental image of this’), mental imagery and visual working memory can share common neural mechanisms in the sensory cortex. In many tasks, participants have to decide for themselves how best to maximize their memory performance. Depending on the ‘mental tools’ at hand, this might be with mental imagery or a propositional strategy. Recent work suggests that imagery strength and the neural networks underlying imagery may play a role in how individuals perform such tasks [37].

The key to unlocking the mechanistic relationship between visual imagery and visual working memory may lie in the individual differences across the population in visual representational strength, physiology, and even anatomy [36]. If a subset of the population tends to utilize imagery to aid memory performance, as the evidence suggests, whereas another subset of people who lack strong imagery utilize a different strategy, collapsing across these two groups could induce inconsistencies in visual working memory data. Separating participants into these groups, based the strength of their imagery, may be a good starting point for gaining clarity on the neural machinery used in visual working memory.

## Graded, System-Wide Activation of Visual Cortex during Mental Imagery

The human visual system is a constellation of functionally distinct areas. These areas are conceived of as being organized in a hierarchy. Activation in areas toward the top of the hierarchy – so-called ‘high-level’ visual areas – is sensitive to changes in the semantic content of visual scenery and is invariant to visual detail. These areas are located in the ventral temporal lobe and representations encoded in the activity of these areas become increasingly abstract toward the anterior pole. Areas toward the bottom of the hierarchy – the ‘early’ visual areas – are located in the occipital cortex and are exquisitely sensitive to visual detail (e.g., retinotopic location, spatial frequency, edges).

Given this organization and the fact that early visual areas both send projections to and receive projections from high-level visual areas, many researchers have predicted that the role of the early visual areas in mental imagery is to flesh out visual detail.

Between 1993 and 2010, at least 20 studies attempted to test this hypothesis by using brain scanning to compare the amplitude of activity in early visual areas during visual mental imagery with the amplitude of activity during perception (or rest). Many studies reported no significant activity above baseline in the early visual cortex during mental imagery [38–45], but a slightly larger number of studies reported significant activity [46–57]. The discrepancy may be explained by differences in experimental factors [58] and variation in the vividness of mental imagery across individuals [55]. Meanwhile, the evidence for activation in high-level areas during imagery is uncontested; studies published over a decade from different groups have shown comparable levels of activity across visual mental imagery and visual perception in high-level visual areas [38,49,59,60]. Although activation in higher areas during mental imagery is more robust than in early visual areas, the vividness of mental images appears to be most tightly coupled to activity in the early visual areas [55]. See Box 1 for other evidence.

However, activation is only part of the picture. Recent studies using **multivariate pattern classifiers (MVPCs)** have shown that the same pattern classifiers that accurately discriminate stimuli by analyzing patterns of activity in visual areas V1 and V2 during perception of simple external stimuli can also discriminate the same stimuli during mental imagery [35,61,62]. This suggests that, although overall levels of activation in V1 during imagery are relatively low, the patterns of activity across imagery and perception in V1 and V2 are similar. This finding again supports the hypothesis of a shared representational format in imagery and perception.

MVPC studies have also revealed similarity in activity patterns across visual perception and imagery in high-level visual areas [59,60,63]. Consistent with activation studies, decoding performance is typically more robust in high-level areas than in early visual areas. Thus, MVPC studies of visual mental imagery support the claim that patterns of activity in perception and imagery become increasingly similar with ascension of the visual hierarchy (see Boxes 1 and 2).

Taken together, the MVPC and activation studies indicate that activity patterns associated with matched external and imagined stimuli begin to resemble one another as early as area V1. The resemblance increases with ascension of the visual hierarchy, although vividness of imagery appears to be most closely associated with early visual areas.

This general picture of how mental imagery engages the visual cortex satisfies many intuitions about how mental images are generated. For example, mental imagery is presumably based on the recall and recombination of memories. Because high-level areas are physically (and synaptically) closer to memory-encoding structures in the medial temporal lobe than are earlier visual areas, it makes sense that the activity patterns associated with perceived and imagined images should more closely resemble one another in high-level than in early visual areas. This may also explain why the semantic aspects of mental images tend to be less ambiguous than visual details (e.g., we can know for certain that we are imagining a zebra and not a horse, even if we are not able to imagine the zebra's individual stripes). Lastly, it makes sense that the parts of the visual system responsible for visual detail should be most closely coupled to the visual vividness of mental images.

## Mental Imagery in Mental Disorders and Their Treatment

In a similar time frame to the burgeoning fundamental mechanistic investigations we have discussed so far, mental imagery has also been found to play a pivotal role in many mental and neurological disorders and their treatments. For example, intrusive, emotional mental imagery causes distress across a range of psychiatric disorders, from post-traumatic stress disorder (PTSD) and other anxiety disorders to bipolar disorder and schizophrenia [64]. However, those psychological therapies primarily based on verbal exchanges have historically neglected imagery, primarily focusing on the patient's verbal thoughts.

After a psychologically traumatic event, a significant proportion of people develop PTSD [65]. PTSD – characterized by re-experiencing the traumatic event through unwanted and recurring intrusive memories and nightmares – provides a hallmark illustration of clinically relevant mental imagery. An example of an intrusive memory is re-experiencing a vivid visual and auditory mental image of the moment a red car hit a child on the sidewalk. Such distressing images may be only fleeting and may occur only a handful of times per week, but their impact can be profound. The patient may avoid reminders of the traumatic event such as cars, children, or walking down a street and may feel a sense of current threat and a racing heart. These imagery-based memories are not a mere epiphenomenon of having PTSD but a cognitive mechanism driving the maintenance of the ongoing clinical disorder [66]. In other words, the intrusive images can strongly affect behavior and physiology.

**Figure 3:**
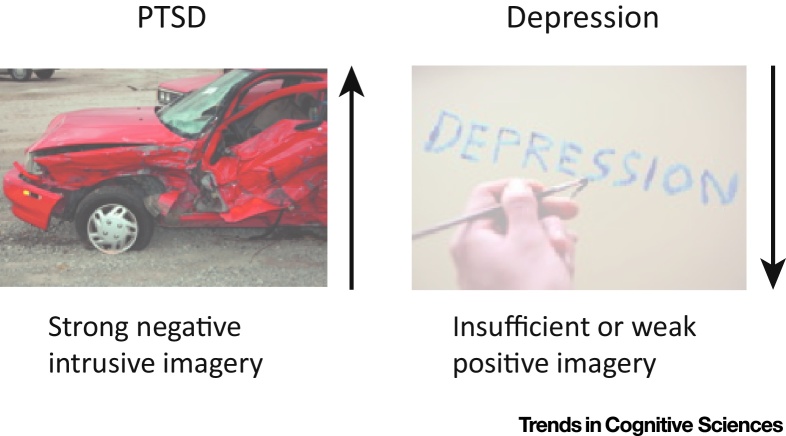
Imagery Is a Key Part of Symptoms in Mental Disorders – From the Intrusive Memories of Trauma in Post-traumatic Stress Disorder (PTSD) to the Lack of Positive Future Imagery in Depression. It presents a cognitive mechanism driving psychopathology, and thus imagery can also be targeted as a process – and harnessed as a tool – in psychological treatment.

Recent years have witnessed an explosion of research suggesting that mental imagery plays a role across a wide range of mental disorders [64,67–69]. Distressing and unwanted emotional imagery has been shown to occur in many mental disorders and the imagery content matches the core concern of people with the disorder. For example, a patient with arachnophobia (fear of spiders) may report imagery of large, hairy spiders with fangs. A patient with obsessive compulsive disorder (OCD) may have images of contaminated grubs boring into his skin and therefore feel dirty, fuelling the behavior of repeated washing. During conversation, a patient with social phobia (fear of public speaking) may experience concurrent imagery of how she appears to her conversational partner, envisioning herself as red and sweating. A patient with bipolar disorder may have future-oriented imagery and ‘flash forward’ to a suicidal act [70]. Conversely, in depression, people can report difficulties imagining a positive future [71] (see Figure 3).

## The Clinical Relevance of Mental Imagery

Given the centrality of intrusive emotional imagery in such a wide variety of mental disorders, a basic understanding of mental imagery could prove instrumental in the development of new treatments. Potentially critical issues include the relative emotional impact of different representational formats, imagery ‘realness’, and the perceived likelihood of imagined events occurring.

Until recently, surprisingly little research had tested the relative impact of picture-like formats versus language-like descriptive formats on emotion; that is, mental imagery versus verbal thought (for an exception see [72]). Recent experiments support the hypothesis that, compared with verbal processing of the same content, mental imagery elicits stronger emotion. For example, in one experiment participants were given negative scenarios with instructions that promoted either verbal processing or mental imagery [73]. Imagery led to a greater increase in anxiety. When presented with positive scenarios, imagery again amplified (positive) emotion [74,75]. Such data are consistent with the finding that emotional memories have more sensory–perceptual features than do non-emotional memories [76].

Other imagery properties are also important. Compared with verbal thoughts of similar content, mental images are rated as more ‘real’ [77]. Many patients report that their imagery ‘feels real’ despite having the knowledge that they are not real, the images have a profound impact on their behavior. The apparent realness of clinical imagery seems to add to its power, influencing not only behavior and emotion but also beliefs. Hallucinations in schizophrenia are defined as mental experiences believed to be external percepts. Both schizophrenia [78] and Parkinson's disease [79] involve involuntary sensory hallucinations. In Parkinson's disease the degree of visual hallucination is well predicted by the sensory strength of an individual's voluntary mental imagery [79].

Repeatedly imagining a future event increases its perceived likelihood of occurrence [80]. This simulation heuristic effect also occurs for anxiety-provoking future events [81], increasing anxiety levels. Conversely, imagining an event that supposedly occurred in the past (even if it did not) inflates a person's confidence that the event actually did occur [82].

Imagined rehearsal of an action influences the likelihood that a person will complete that action [83]. Although such promotion of a behavior might be useful when actions are desired such as in sports psychology, one can see how its consequences can be maladaptive in psychopathology; for example, by increasing washing behavior in OCD. Similarly, imagery of a desired substance may contribute to cravings and thereby drive addictive behaviors [84,85]. In depression, imagining suicidal acts may even increase the risk of suicide [86]. Conversely, impaired ability to simulate positive future events is related to depression [87,88] (a disorder characterized by pessimism) whereas trait optimism is associated with greater ability to mentally simulate positive future events [119].

## Mental Imagery in Clinical Treatments

These intriguing results on the emotional and behavioral impact of mental imagery offer insights into the development of new treatments for anxiety disorders. It is difficult to treat problematic emotional imagery with purely verbal discussion in therapy: to reduce imagery symptoms effectively, therapeutic techniques should include an imagery-focused component. Mental imagery techniques are currently used in some evidence-based treatments. For example, cognitive behavioral therapy (CBT) often includes ‘imaginal exposure’, which involves having the patient repeatedly imagine the feared object or context (e.g., contaminated hands) until his or her anxiety level subsides [89]. Imaginal exposure is a key technique, used across anxiety disorders.

Another technique, ‘imagery rescripting’ [68], aims to transform the imagery content. For example, in social phobia the negative outcome of mental imagery (e.g., performing badly) is changed to a new, more adaptive image such as performing competently [90]. ‘Systematic desensitization’ uses gradual exposure to images of feared objects or situations, whereby the imagery is paired with an incompatible response to the fear – such as physical relaxation – until the image no longer evokes negative emotion [91]. A form of therapy called ‘eye movement desensitization and reprocessing’ (EMDR) promotes lateral eye movements during the recall of emotional memories; this technique appears to dampen the vividness and emotionality of imagery [92].

These imagery-focused therapeutic techniques reduce the powerful impact of dysfunctional imagery on emotion and/or reduce the frequency of associated intrusive imagery. It is noteworthy that imagery-focused CBT, as reviewed in clinical guidelines [120] has the strongest documented impact on treating PTSD and social phobia, with some trials showing success rates of up to 75%.

## Future Mental Imagery Treatments

How might mental imagery research lead to future treatment innovations? First, we can import existing imagery techniques to clinical areas where imagery has been neglected. For example, treatments for bipolar disorder have shown little improvement since the discovery of lithium many decades ago. Perhaps advances can be made by leveraging the fact that people with bipolar disorder show high spontaneous use of imagery and intrusive imagery [70,93]. By considering the possible role of imagery in this disorder, new treatments could be devised by importing imagery techniques from those used to treat anxiety disorders. Another example is addressing hopeless, pessimistic future orientation by training patients with depression to generate more adaptive mental imagery and simulate future positive events. An initial randomized controlled trial including computerized positive imagery training in depressed patients showed some promising results [94] (though see also [71]), requiring further research.

Second, basic science studies of mental imagery may inform the development of new imagery treatment techniques by focusing on the depictive, pictorial format of imagery itself. For example, as discussed above, concurrent perception may interfere with image generation [2,12,95]. This finding is consistent with the fact that strategically applied visual tasks, such as the computer game Tetris, performed soon after an experimental trauma (in the time window for memory consolidation), reduce the frequency of intrusive images [96]; this technique has recently been extended to reconsolidation [97]. Such findings may open ways to prevent the accumulation of intrusive images, which is important because we need preventative treatments for PTSD [98]. Linking studies of emotional imagery with neural mechanisms may also be useful [99,100].

Overall, discoveries about mental imagery can contribute to our understanding of the cognitive and neural mechanisms underlying psychopathology and of which mechanisms to target in improving treatments [101]. Even the best treatments do not work for everyone and effective treatments are not yet available for all mental disorders. Science-driven mental imagery treatment techniques could greatly help and may even offer treatment innovations that look little like traditional talking therapies. Such treatments would capitalize on the principle that imagery involves a depictive format with its own set of properties [3] (Box 2).

## Concluding Remarks

The many new methods touched on here not only offer new mechanistic insights into mental imagery but also offer new tools for future research. Recent work has demonstrated how imagery can ‘stand in’ for an afferent visual representation of an external stimulus. Specifically, mental images seem to behave much like weak versions of externally trigged perceptual representations. Functional brain imaging work supports the behavioral evidence by demonstrating that common sets of neural structures are employed during both events. Further, both representations seem to be encoded using a common set of basic visual features, which in many visual areas are organized topographically.

An increasingly important component of imagery research now and in the future is the translation of the fundamental science into the clinic. Clinical research shows that many different mental disorders involve symptomatic imagery, and incorporating imagery into behavioral treatments is proving beneficial. Bridges from fundamental research to emotional imagery will be critical for the systemic understanding of mental representations in dysfunction. Similarly, the characteristics and function of mental representations in everyday cognition will help form a fuller understanding of human mental events.

The main functions of mental imagery include simulating possible future scenarios and ‘reliving’ past experiences [83,102,103]. From this perspective, imagery should perhaps be studied not only in its own right but in many types of cognitive tasks. Beyond visual working memory, we know that imagery plays a role in affective forecasting [104], eye witness memory [105], making certain moral decisions [106], prior expectation templates to aid in predictable visual tasks [107], and facilitating emotion [108]. Mental simulations are now used to detect consciousness in vegetative state patients [109] and can be decoded using brain imaging during the early stages of dreams [110]. One interesting proposition is that all forms of cognition involve modality-specific mental simulations, known as embodied or grounded cognition [111]. Such theories imply that imagery plays a functional role in all cognitive events. It is exciting to begin to see the detailed, ubiquitous, and multifaceted role imagery plays in our everyday lives, both in function and dysfunction.

Outstanding Questions How do perception and mental imagery differ? There are clear phenomenological and epistemological differences between external perceptual and mental images, and patterns of activity measured during imagery and perception of the same stimulus are not identical. A first step toward answering this question will be to discover whether imagery-induced neural activity patterns are simply weaker or noisy versions of the activity during the perception of matched external stimuli or whether they encode systematically distorted representations.

Are individuals able to exploit the differences between mental imagery and perception?

How does mental imagery differ from other forms of top-down activity? Visual perception is heavily influenced by working memory, attention, and expectation. Clearly mental imagery is related to these disparate cognitive phenomena, but more work is needed to elucidate the networks and patterns of neural activity that distinguish mental imagery from these and other modes of cognition and perception.

Can mental imagery be involuntary, as clinical theory proposes? Or do individuals simply lack conscious awareness of the voluntary process (e.g., have poor metacognition)? The type of imagery prevalent in many mental disorders is typically described as involuntary, or not under the individual's control (see the clinical section). Little is known about the mechanisms that distinguish voluntary and involuntary imagery.

Can mental images be generated non-consciously?

What functional mechanisms dictate individual differences in imagery strength?

There are many examples of visual illusions that create a conscious visual experience without a direct stimulus. Might the involuntary nature of such phantom perceptual experience offer a novel way to study the involuntary elements of imagery?

## Disclaimer Statement

The views expressed are those of the authors and not necessarily those of the NHS, the NIHR, or the Department of Health. Funding to pay the Open Access publication charges for this article was provided by the United Kingdom Medical Research Council.

## Box 1 Brain Damage Studies

Many studies have related measures of imagery to damage in particular parts of the brain. For example, in one study researchers [112] asked patients with unilateral neglect to imagine standing at either end of a well-known piazza in Milan, Italy. The primary symptom of unilateral neglect is that when patients look at a perceptual scene in front of them they tend to neglect one side of space. When asked to describe the piazza in their mind's eye, the patients described landmarks on one side of the square only. To ensure that this was not due to memory deficits, patients were asked to imagine the piazza from the opposite vantage point; the patients could describe the details of the previously neglected side, but now neglected the other side. This result was taken as evidence that imagery and perception share common neural processes at the level of attentional deployment.

Damage to the early visual cortex has also been diagnostic in the role of area V1 in imagery generation. In one study researchers were able to test the size of visualized objects both before and after a unilateral occipital lobe resection for epilepsy treatment [113]. Using a particular method that involves imagining an object and then walking toward it in the mind's eye until the object fills the entire visual field, and then reporting the distance between the individual and the object, researchers are able to infer the maximal image size. Here they found that after surgery the patient's maximal image size shrunk in the horizontal dimension compared with the image size before surgery.

Such data suggest that area V1 plays a functional role in visual mental imagery. However, other studies have demonstrated that it is possible to have intact and even vivid mental imagery, both behaviorally and when assessed using brain imaging, despite near-complete blindness due to cortical grey matter damage in the calcarine sulcus (V1) [114]. Hence, damage to V1 will impair mental images but, even with V1 almost completely gone, mental imagery remains possible (Box 2).

Thus, the results from studies of patients with brain damage are consistent with the results from fMRI and behavioral studies noted in the text: early visual areas can contribute to imagery, but other areas also play key roles. This inference is consistent with the idea that mental images, like visual percepts, rely on representations that are collaboratively constructed by visual areas at all stages of the visual processing hierarchy.

## Box 2 Primary Visual Cortex and Mental Imagery

During visual perception, area V1 is distinguished both by its anatomical location and by the visual features that are encoded in its activity. This area is anatomically privileged because it is a gatekeeper of retinal information into the cortex. It receives more direct connections from the lateral geniculate nucleus than any other part of the visual cortex. However, during mental imagery its proximity to the retina does not make it special. The source of mental imagery is unknown, but it is likely that memory-encoding structures in the medial temporal lobe (MTL) and executive structures in the prefrontal cortex are critical. In addition, area V1 is distinguished by its representation of low-level visual features. Feed-forward models of perception treat these low-level features as the building blocks of object representation. However, feedback models treat them not as foundational for constructing representations of objects but as a tool for error-checking predictions about what objects are present in the immediate environment [115].

The anatomical importance of V1 during mental imagery may be derived from its topographical organization, which allows it to make explicit and accessible geometric properties that are only implicit in representations stored in long-term memory. In other words, the role of V1 in imagery may be determined by the kinds of inferences it allows to be drawn from a mental image. For example, if we want to infer whether a German shepherd dog has pointed or floppy ears [116] we may need to invoke a V1-like representation as a component of our mental image. If we simply want to infer whether an elephant is bigger than a mouse, it may suffice to invoke representations in any one of the many visual and/or parietal areas that are topographically mapped. This idea is perfectly consistent with findings that imply that the extent of V1 activation during mental imagery is task dependent.

In addition, the role of V1 in imagery is likely to vary enormously across individuals. Area V1 may make very different contributions to mental images for different people, depending on how important its representations are to the way in which each person imagines objects and/or scenes [117]. Recent research has documented that the size of area V1 predicts the sensory strength and precision of visual imagery [24]. Such relationships dovetail nicely with capacity limitation theories that propose an interaction between the content and anatomical restrictions due to the 2D layout of structures like V1, which support the representations [118].

## Glossary

Binocular rivalry: a visual phenomenon in which two different patterns are presented, one to each eye; the patterns compete for perceptual dominance, such that during continuous viewing awareness alternates between the two patterns.
Low-level visual features: in this context refers specifically to perceptual visual features such as color, spatial orientation, contrast, and spatial frequency; features of visual stimuli that are largely processed by the early visual cortex.
Multivariate pattern classifiers (MVPCs): also referred to as multivariate decoding; in fMRI, typically the use of spatial patterns (many voxels) to make a prediction or classification regarding some perceptual, cognitive, or behavioral state. The activation of multiple voxels from fMRI data is used as a pattern rather than averaging over a region of interest.
Retinotopic: refers to the mapping of information from the layout of the retina in the eye to the visual cortex. Cells in the visual cortex respond to stimulation of a specific part of visual space, such that two adjacent cells will respond to two adjacent stimuli hitting the retina.
Voxel: in fMRI, the smallest unit of measured data.
